# Natural Extract Combination Modulates Intestinal Barrier and Hepatic Cholesterol via the Gut–Liver Axis In Vitro

**DOI:** 10.3390/pharmaceutics18030328

**Published:** 2026-03-05

**Authors:** Francesca Uberti, Simone Mulè, Francesca Parini, Matteo Musu, Rebecca Galla

**Affiliations:** 1 Department for Sustainable Development and Ecological Transition, University of Piemonte Orientale (UPO), Piazza Sant’Eusebio 5, 13100 Vercelli, Italy; simone.mule@uniupo.it (S.M.); 20049586@studenti.uniupo.it (M.M.); 2 Noivita Srls, Spin Off, University of Piemonte Orientale (UPO), Strada Privata Curti n. 7, 28100 Novara, Italy; francescaparini00@gmail.com (F.P.); rebecca.galla@noivita.it (R.G.)

**Keywords:** gut-liver axis, cholesterol homeostasis, hypercholesterolemia, intestinal barrier function, nutraceutical approach, botanical extracts

## Abstract

**Background/Objectives:** The gut–liver axis plays a central role in cholesterol homeostasis, linking intestinal absorption, microbial metabolites, and hepatic lipid regulation. Dysregulation of this axis contributes to hypercholesterolemia and cardiometabolic risk, beyond classical cholesterol synthesis pathways. This study evaluated a novel multi-botanical formulation (MIX) that combines *Gastrodia elata*, Black Garlic, *Primula veris*, and *Emblica officinalis* (AMLA) to integrate modulation of cholesterol metabolism through intestinal and hepatic mechanisms. **Methods:** Individual extracts were chemically characterised for polyphenols, flavonoids, polysaccharides, S-allyl-L-cysteine (SAC), and tannins. Caco-2 cells were treated with varying doses to determine optimal concentrations and for viability, transepithelial electrical resistance, and permeability analysis. Supernatants post-intestinal passage were applied to HepG2 cells under high-glucose conditions to assess viability, oxidative stress, SRC/ERK-MAPK signalling, cholesterol synthesis (HMGR), LDL uptake, PCSK9–LDLR–SREBP-2 axis, and bile acid production. **Results:** MIX enhanced intestinal barrier integrity (TEER, tight junctions, permeability) and preserved cell viability compared with single extracts. In HepG2 cells, MIX demonstrated synergistic effects: it reduced HMGR expression by 83–90% relative to individual extracts, increased LDLR expression by 43–97%, suppressed PCSK9 by up to 92%, and lowered total cholesterol and LDL uptake more effectively than RYRF. MIX also amplified bile acid production and free cholesterol excretion, indicating improved hepatic clearance pathways. SRC and ERK-MAPK signalling were favourably modulated, supporting hepatocyte survival under metabolic stress. **Conclusions:** The multi-botanical formulation exerts complementary and synergistic effects on intestinal absorption and hepatic cholesterol regulation, integrating suppression of cholesterol synthesis, enhanced LDL clearance, and stimulated elimination via bile acids. These findings highlight the potential of the MIX formulation to modulate metabolically induced cholesterol dysregulation, supporting further in vivo and clinical investigation.

## 1. Introduction

The increasing prevalence of dyslipidemia and the shortcomings of single-molecular target-based cholesterol-lowering techniques have sparked a resurgence of interest in multi-target solutions that can physiologically and comprehensively modify lipid homeostasis [1,2]. The intestinal lipid absorption, epithelial barrier function, bile acid-mediated signalling, and hepatic metabolism work together to determine plasma low-density lipoprotein (LDL) cholesterol levels in this setting, making the gut–liver axis a key platform of metabolic control [3].

The gut actively and decisively regulates cholesterol metabolism, serving not only as an absorption site but also as a sensor and regulatory organ [4]. To control lipid flow and stop pro-inflammatory compounds from moving into the portal circulation, the integrity of the intestinal barrier must be preserved. Dyslipidemia, low-grade systemic inflammation, and liver dysfunction have all been linked to changes in barrier function and gut microbiota composition, which greatly contribute to the dysregulation of lipid homeostasis [5]. Bidirectional communication along the gut–liver axis, facilitated mostly by bile acids and their receptors, enables the liver to integrate signals from the stomach and modify cholesterol production, LDL receptor expression, and lipoprotein processing. In this model, the liver is not an isolated compartment, but rather a regulatory node that responds dynamically to intestinal inputs, strengthening the idea of systemic and coordinated control of lipid metabolism [6].

Nutraceutical strategies that are exclusively oriented toward modulating endogenous cholesterol synthesis appear partial [7]. Fermented red rice (RYRF) is an emblematic example of this approach, traditionally used for its presence of monacolin K, a direct inhibitor of HMG-CoA reductase (HMGR) with a mechanism of action superimposable on that of statins [8]. Although effective in reducing cholesterol levels, this mechanism is limited and is associated with significant clinical and regulatory concerns. It has been well documented that taking monacolins from RYRF can induce adverse effects analogous to those of statins, including muscle and liver events, even at relatively low doses [9]. Consistently, the European Food Safety Authority (EFSA) concluded that it is not possible to identify a level of monacoline intake from RYRF that is free of potential health risks, leading to increasingly stringent restrictions on their use [9,10]. Added to this are problems related to the variability of monacoline content, the poor standardisation of commercial products and the risk of contamination by citrinin, a nephrotoxic mycotoxin frequently found in fermented products [11].

In this setting, the search for novel botanical extracts, either alone or in combination, provides a viable strategy to modulate elevated plasma cholesterol levels by targeting critical metabolic pathways, rather than the direct inhibition of HMGR characteristic of monacolins [12,13]. Botanical extracts may promote more physiological metabolic remodelling than standard treatments by enhancing biliary cholesterol excretion, modulating SREBP-2 expression, and regulating LDL clearance through PCSK9 and LDLr [14]. The botanical extracts employed in this study include AMLA (*Emblica officinalis*), Black Garlic (*Allium sativum* fermented), *Gastrodia elata*, and *Primula veris*, which were chosen based on their functional complementarity along the gut–liver axis.

AMLA is the most investigated and described component. It contains polyphenols, hydrolyzable tannins, and ascorbic acid, and studies have shown that AMLA extracts can reduce lipid absorption and modify the gene expression of PCSK9, LDLr, and SREBP-2, resulting in antioxidant and hepatoprotective actions [15,16]. In vivo evidence in animal models verifies the capacity of AMLA extract to lower cholesterol levels and improve the liver lipid profile, supporting the translatability of cellular data [16]. The other extracts of the formulation have complementary characteristics that enhance AMLA’s hepatic and intestinal activity. Black Garlic, through the content of organosulfur species such as S-allyl-cysteine (SAC) and water-soluble antioxidant compounds, supports LDL clearance and reduces hepatic oxidative stress, also in in vitro studies in HepG2 cells [17,18]. *Gastrodia elata* contributes to liver protection and the reduction in cellular metabolic stress through its phenolic compounds and gastrodin, supporting liver cell function under lipid-loading conditions [19,20]. Finally, *Primula veris* is also the least studied in this context, but some of its components, flavonoids and triterpene saponins [21], in common with other extracts described, can promote faecal cholesterol excretion by binding bile salts, indirectly stimulating the hepatic conversion of cholesterol into bile acids and integrating the metabolic action of the other extracts [22].

Considering this evidence, following an accurate chemical characterisation of the botanical extracts under examination, the present study aims to evaluate in vitro using a Transwell^®^ Caco-2 and HepG2 model, the anticholesterolemic and hepatoprotective effects of a formulation based on AMLA, *Gastrodia elata*, *Primula veris* and Black Garlic, specifically designed to combine chemically distinct phytochemical profiles in order to explore potential multi-target interactions along the gut–liver axis. The inclusion of *Primula veris*, an extract scarcely investigated in this context, provides novel insight into its potential modulatory role within a combined botanical preparation. The activity of the formulation was compared with that of a standardised monacolin-containing RYRF, used as a mechanistic reference compound to enable comparative pathway analysis. Indeed, RYRF was a relevant comparator for its content of monacolin K, a natural HMG-CoA reductase inhibitor, which provides a well-characterised benchmark for assessing the lipid-lowering potential of botanical extracts.

HepG2 cells are widely used as an in vitro model to study lipid metabolism and to evaluate the effects of nutraceuticals on oxidative stress and inflammation [23]. They were exposed to high glucose (D-glucose 30 mM) conditions to simulate a state of hepatic lipid stress. The samples were so evaluated in an in vitro condition which induces metabolically driven cholesterol dysregulation rather than primary hypercholesterolemia.

This study was designed to provide mechanistic insight, under controlled in vitro conditions, into whether a multi-target nutraceutical strategy directed at the gut–liver axis may offer potential functional advantages. To this end, both safety aspects, in terms of maintaining the integrity of the intestinal barrier, and bioactive effects at the hepatic level were evaluated, including hepatocyte protection, modulation of cholesterol metabolism and regulation of the main PCSK9–LDLR–SREBP-2 pathways, central for the control of LDL clearance and lipid synthesis, demonstrating that the combined formulation exerts synergistic effects, particularly at the liver level.

## 2. Materials and Methods

### 2.1. Characterisation of Extracts

The extracts were of commercial origin and were received as pre-processed, standardised preparations. Therefore, no direct botanical authentication or voucher specimens were available to the authors. According to the supplier, the extracts were obtained through standardised procedures designed to preserve the main bioactive compounds and ensure reproducibility and stability. Briefly, AMLA dried fruits were extracted with a water–ethanol mixture, followed by filtration, concentration, and drying to yield a powder rich in phenolic compounds. *Primula veris* aerial parts were extracted in water at a 4:1 solvent-to-plant ratio, then filtered, concentrated, and freeze-dried to preserve thermolabile flavonoids and other water-soluble metabolites. Garlic bulbs were extracted in water or aqueous ethanol, filtered, concentrated, and dried to obtain a powder enriched in sulfur-containing compounds. *Gastrodia elata* rhizomes underwent heat treatment (boiling or steaming) to facilitate the release of water-soluble polysaccharides and polyphenols, followed by filtration and drying into a standardised powder.

The phytochemical characterisation conducted in this study employed a targeted analytical approach and should therefore be regarded as semi-quantitative. Quantification was restricted to selected marker compounds, including SAC and other predominant constituents, based on available reference standards. Spectrophotometric assays (Folin–Ciocalteu for total polyphenol content, TPC, and AlCl_3_ for total flavonoid content, TFC) were used to quantify overall phenolic and flavonoid classes as comparative functional indices, whereas HPLC analysis was performed for the identification and quantification of specific individual compounds. The combined use of these approaches was intentional, as they provide complementary class-level and molecular-level information rather than interchangeable measurements.

The methodologies used do not provide complete metabolomic coverage, nor do they enable full identification and quantification of minor or structurally related secondary metabolites. Consequently, the analysis offers standardisation of the extract and an evaluation of the relative composition but does not constitute an exhaustive qualitative-quantitative phytochemical profiling of the studied formulations.

#### 2.1.1. Determination of Total Polyphenol Content

A Folin–Ciocalteu-based colourimetric assay was employed to determine TPC, with slight modifications from previously described methods. In brief, an aliquot of the sample solution was mixed with Folin–Ciocalteu reagent and incubated for 3–5 min at room temperature. After alkalinising with aqueous sodium carbonate, the mixture was kept in the dark at room temperature for 30–60 min to develop colour. A UV–Vis spectrophotometer (Infinite 200 Pro MPlex plate reader, Tecan, Männedorf, Switzerland) measured the absorbance of the solution at 760 nm. A calibration curve was generated using gallic acid as the external standard over an appropriate concentration range. TPC was expressed as % (*w*/*w*, dry extract). Measurements were performed in triplicate and are presented as mean ± standard deviation (SD).

#### 2.1.2. Bate–Smith Tannin Analysis by Acid Hydrolysis

The Bate–Smith procedure measures total tannins by acid hydrolysing condensed tannins to produce coloured anthocyanidins. A sample is mixed with a 95:5 *v*/*v* n-butanol–HCl reagent with ferric ions as a catalyst, then boiled at 95–100 °C for 40–60 min. After cooling, the absorbance at 550 nm is measured using a reagent blank. Tannin is quantified either by a standard component or absorbance units per gram of extract, with data expressed as equivalents of a reference standard via a standard curve (10–100 µg/mL). The method is linear within this range (R^2^ > 0.99). At least three experiments were performed, and data are presented as mean % (*w*/*w*, dry extract) ± SD.

#### 2.1.3. Quantification of Total Polysaccharides by Phenol-Sulfuric Acid Method

The total polysaccharide content was determined using the phenol–sulfuric acid colourimetric assay [24]. Briefly, a portion of the *Gastrodia elata* extract was dissolved in purified water and diluted to ensure measurements fell within the assay’s linear range. Next, 1.0 mL of this solution was mixed with 1.0 mL of a 5% (*w*/*v*) phenol solution, followed by the rapid addition of 5.0 mL of concentrated sulfuric acid along the side of the test tube to promote thorough mixing. The mixture was then left to stand at room temperature for 30 min to allow complete colour development. The absorbance of the resulting solution was measured at 490 nm using a UV–Vis spectrophotometer, with a reagent blank as the reference. The amount of polysaccharide was determined from a calibration curve generated with D-glucose as the standard. All experiments were conducted in at least triplicate. Results are presented as the mean percentage (*w*/*w*, on a dry extract basis) ± SD.

#### 2.1.4. SAC and Tannins Analyses Employing HPLC Methods

Black Garlic extract’s SAC was analysed as previously described [25] using an HPLC-MS/MS system (TSQ Quantum Access Max, Thermo Scientific Waltham, MA, USA) with an autosampler and nitrogen gas generator. Mass spectrometry settings included positive-mode electrospray ionisation, a scan rate of 0.5 spectra/s, 10 eV collision energy, and SRM with precursor ions at *m*/*z* 162 and product ions at *m*/*z* 73 and 145. Samples (5 µL) were injected into a Zorbax^®^ C18 column (250 × 4.6 mm, 5 µm; Agilent Technologies, Santa Clara, CA, USA). A gradient of solvent A (0.1% formic acid in water) and solvent B (acetonitrile): 0–7 min, 20% B; 7–14 min, 100% B; 14–17 min, 100% B; 17–25 min, 1% B. Xcalibur 2.1 (Thermo Scientific) was used to calculate SAC concentrations, which showed a linear calibration curve (R^2^ ≥ 0.99). Instead, tannins in AMLA extract were quantified using HPLC-DAD. Briefly, samples were dissolved in aqueous methanol and injected (20 µL) onto a C18 reversed-phase column (250 × 4.6 mm, 5 µm) and eluted with a gradient of solvent A (0.1% formic acid in water) and solvent B (acetonitrile). Detection was performed with a diode array detector at 280 nm, and gallic acid was used as the reference standard.

At least three measurements were performed; results are expressed as mean % (*w*/*w*, on a dry extract basis) ± SD.

#### 2.1.5. Determination of Total Flavonoid Content

A colourimetric method was used to measure TFC [26]. Briefly, 1 mL of *Primula veris* extract (0.1 mg/mL) was mixed with 4 mL water in a 10 mL flask. Then, 3 mL of 5% NaNO_2_ was added. After 5 min, 0.3 mL of 10% AlCl_3_ was added. One minute later, 2 mL of 1 mM NaOH was added. The flask was topped up with distilled water, mixed, and absorbance was recorded at 510 nm. Measurements were performed in triplicate or more, and results are expressed as mean % (*w*/*w*) relative to dry extract ± SD.

### 2.2. Agents’ Preparation

The substances tested included: AMLA (12:1 fruit extract in Dyno patent n° 102024000003076 and PCT/IB2025/051594), Black Garlic extract (0.66% SAC), *Primula veris* extract (4:1 leaf extract, also called Primula), *Gastrodia elata* extract (rhizoma extract; 10.9% polysaccharides), and RYRF extract (*Oryza sativa* L. extract; 5.2% monacolines) were freshly prepared. All plant extracts were supplied as powdered, ready-to-use materials by Nutra Futura s.r.l. (Legnano, Italy). Quality control and chemical characterisation were performed to confirm the main active components in accordance with the manufacturer’s specifications, as described in Section 2.1.

Botanical extracts were diluted in DMEM (without phenol red), without foetal bovine serum, and treated with antibiotics and L-glutamine before each stimulation. However, *Gastrodia elata* was evaluated at 100 µg/mL as described in earlier in vitro research [27]. All other doses used in dose–response tests were selected based on previous literature on hepatic cell models [28,29,30,31,32]. AMLA (25, 50, 100 μg/mL), Black Garlic (10, 25, 50 μg/mL), *Primula veris* (50, 100, 125 μg/mL), and RYRF (8, 80, 160 μg/mL) were the quantities assessed.

The composition of the multibotanical MIX was defined based on the individual extract concentrations determined in preliminary dose–response experiments to ensure experimental consistency and combined in an equimolar design guided by efficacy data to maximise synergistic interactions while preserving cell viability for mechanistic evaluation in vitro.

### 2.3. Cell Cultures

The human Caco-2 cell line, originating from a Caucasian colon adenocarcinoma, was obtained from the American Type Culture Collection (ATCC; Manassas, VA, USA). Cells were cultured in Dulbecco’s Modified Eagle Medium Advanced formulation (DMEM-Adv; Thermo Fisher Scientific, Rodano, MI, Italy) supplemented with 10% foetal bovine serum (FBS; Merck Life Science, Rome, Italy), 2 mM L-glutamine, and 1% penicillin–streptomycin (Merck Life Science, Rome, Italy). Cultures were maintained at 37 °C in a humidified atmosphere containing 5% CO_2_. The Caco-2 cell line is widely accepted by regulatory authorities, including the Food and Drug Administration (FDA) and the European Medicines Agency (EMA), as a validated in vitro model for predicting intestinal absorption, metabolism, and oral bioavailability [33,34]. For the experimental procedures, the cells were seeded differently. For dose–response experiments, cells were seeded at a lower density (1 × 10^4^ cells per well) in 96-well plates, while for safety analyses, integrity and permeability 2 × 10^4^ cells were seeded into 24-well plates equipped with 6.5 mm Transwell^®^ inserts containing a 0.4 μm pore polycarbonate membrane (Corning Costar, New York, NY, USA). Prior to stimulation, cells were incubated for 8 h in phenol red-free DMEM-Advanced medium–supplemented with 0.5% FBS, 2 mM L-glutamine, and 1% penicillin–streptomycin (Merck Life Science, Rome, Italy) [35].

HepG2 cells, a human hepatocellular carcinoma-derived cell line (ATCC, Manassas, VA, USA), were cultured under controlled conditions at 37 °C in a humidified atmosphere containing 5% CO_2_ [36]. Cells were maintained in Dulbecco’s Modified Eagle Medium (DMEM) supplemented with 10% FBS, 2 mM L-glutamine, and 1% penicillin–streptomycin (Merck Life Science). To ensure reproducibility, only cultures reaching 90–95% confluence were used for experimental procedures [37]. For all experimental analyses, 3.5 × 10^4^ cells were seeded in 24-well plates fitted with 6.5 mm Transwell^®^ inserts. The establishment of a differentiated and intact monolayer was verified by monitoring trans-epithelial electrical resistance (TEER), which reached approximately 486 Ω·cm^2^ [38]. During the experimental phase, cells were cultured in medium supplemented with 30 mM glucose (Merck Life Science, Milan, Italy). Controls were maintained either under standard glucose conditions or in high-glucose medium (30 mM) without treatment, serving as reference controls [36,39]. HepG2 cells were exposed to 30 mM D-glucose, a commonly used high-glucose condition, to promote the accumulation of intracellular cholesterol and metabolism dysregulation [40,41].

### 2.4. Experimental Protocol

The study was structured in three main phases. In the first step, after fully characterising the chemical composition of each botanical extract, a preliminary 1–6 h dose–response study was carried out on Caco-2 cells (except for *Gastrodia elata* as described in Section 2.2) to identify concentrations of Black Garlic, AMLA, *Primula veris*, and RYRF that did not affect cell viability. The 3-(4,5-dimethylthiazol-2-yl)-2,5-diphenyltetrazolium bromide (MTT) assay was used to determine safe concentrations for use in subsequent experiments in single and in combination (MIX: AMLA + Black Garlic + *Gastrodia elata* + *Primula veris*). Extract concentrations were chosen to ensure cell viability and avoid cytotoxicity, allowing reliable assessment of metabolic effects. Higher doses were not used to prevent confounding effects from cell stress. These ratios served as a reference framework for the integrative LC–MS/MS chemical characterisation of the MIX (Appendix A).

In the second phase of the study, the individual botanical extracts, MIX and RYRF were examined, always in the period 1 h–6 h, on the intestinal barrier model on Transwell^®^. The multibotanical MIX was formulated from concentrations identified in preliminary dose–response experiments and combined in an equimolar ratio to maximise synergy while maintaining cell viability. The analysis evaluated cellular viability (MTT assay) and the integrity of cellular barriers by measuring TEER. Additionally, analysis of proteins’ tight junctions (TJ) levels (claudin-1; occludin and ZO-1) through ELISA kits and the permeability of the samples along the intestinal barrier in vitro were evaluated using a fluorescent probe (fluorescein). At the conclusion of the treatment period, the supernatant from each sample in the basolateral compartment of the intestinal barrier model on Transwell^®^ inserts was collected and employed as a stimulus for the in vitro hepatic model. This methodology aimed to recreate a functional gut–liver axis under controlled experimental and cellular conditions, facilitating the study of inter-organ interactions and metabolic signalling between the intestine and liver.

Thus, the third phase focused on the liver, using a high-glucose HepG2 model to mimic metabolic stress conditions associated with altered cholesterol metabolism in vitro.

Before stimulation, HepG2 cells were synchronised overnight in phenol-red-free DMEM with foetal bovine serum, penicillin/streptomycin, L-glutamine, and sodium pyruvate. They were then exposed to 30 mM high-glucose medium to mimic hyperglycemia [36]. Cells were treated with intestinal supernatants and RYRF at 8 μg/mL for 24 h to assess cholesterol metabolism, measuring total and free cholesterol, LDL uptake, and bile acid synthesis. Cytotoxicity was checked with MTT and ROS, and liver cell integrity with ELISA assays for pSRCTyr^529^/SRC and ERK/MAPK. Key cholesterol pathways were studied by ELISA and Western blot for proteins like HMGR, SREBP-2, PCSK9, and LDLR.

### 2.5. Cell Viability (MTT Assay)

Cytotoxicity was evaluated using a standard MTT assay [42]. Briefly, both cell lines were stimulated and incubated with 1% MTT solution (Merck Life Science, Milan, Italy) in DMEM for 2 h at 37 °C. An equal volume of MTT solubilisation solution was then added to dissolve the purple formazan crystals. To determine cell viability, absorbance at 570 nm was measured with an Infinite 200 Pro MPlex plate reader (Tecan, Männedorf, Switzerland), using a reference reading at 690 nm. Data are presented as mean percentage ± SD relative to untreated controls (0%), based on five separate, triplicate measurement trials.

### 2.6. In Vitro Model of Intestinal Barrier

TEER was measured using an EVOM3™ Volt/Ohm Meter with STX2 electrodes (World Precision Instruments, Sarasota, FL, USA) to monitor the formation of a polarised and intact Caco-2 cell monolayer and the development of paracellular pathways. Only monolayers with TEER readings above 400 Ω·cm^2^ on day 21 were tested for permeability [43]. Before adding compounds, the apical medium was adjusted to pH 6.5 to simulate the small intestine lumen, while the basolateral medium was set to pH 7.4 to imitate systemic circulation [44]. Cells were exposed to test compounds for 1–6 h. Paracellular transport was evaluated using fluorescein sodium salt tracer (0.04%, 376.27 Da, Santa Cruz, CA, USA) [27]. The probe was added to the apical compartment of differentiated Caco-2 monolayers, and its appearance in the basolateral compartment over time was measured to evaluate apparent apical-to-basolateral permeability. In detail, after 40 min incubation at 37 °C, tracer passage was quantified by fluorescence (excitation 490 nm, emission 514 nm) using an Infinite 200 Pro MPlex spectrophotometer (Tecan, Männedorf, Switzerland). The permeation rate was calculated according to the previously described equation [35]:

J = Jmax [C]/(Kt + [C])

where

- Jmax: the maximum permeation rate;
- [C]: the initial concentration of fluorescein;
- Kt: the Michaelis–Menten constant.

The results are displayed as mean ± SD (%) and include cell-free negative controls to remove the effects of the Transwell^®^ membrane.

### 2.7. Analysis of Human TJ Proteins

To measure occludin (OCLN), claudin-1, and ZO-1 (TJP1) using specific ELISA kits (MyBiosource, San Diego, CA, USA; Cusabio Technology, Houston, TX, USA), Caco-2 cell lysates were prepared following the manufacturer’s instructions. A Tecan Infinite 200 Pro-MPLex spectrophotometer (Männedorf, Switzerland) was employed to detect absorbance at 450 nm. Standard curves were used to determine analyte concentrations, ranging from 0 to 1500 pg/mL for occludin and from 0 to 1000 pg/mL for ZO-1 and claudin-1. The results are based on five separate experiments, each performed in triplicate, and are presented as percentages relative to untreated controls, which are set at 0%.

### 2.8. ROS Production

Superoxide anions were quantified using a validated spectrophotometric assay [45], which measures cytochrome C reduction at 550 nm with a Tecan Infinite 200 Pro MPlex reader (Männedorf, Switzerland). The O_2_^−^ release rate was expressed as the mean ± SD of nanomoles of reduced cytochrome C per microgram of protein, shown as a percentage relative to control, based on five experiments in triplicate.

### 2.9. ERK/MAPK Activity

ERK/MAPK pathway activation was quantified in HepG2 lysates using the InstantOne™ ELISA kit (Thermo Fisher Scientific, Milan, Italy) [46]. ELISA plates coated with specific antibodies were treated with 50 µL of protein extracts in lysis buffer for 1 h at room temperature with shaking. A detection reagent was added for 20 min, then a stop solution was used to end the reaction. Absorbance was measured at 450 nm using an Infinite 200 Pro MPlex spectrophotometer (Tecan, Männedorf, Switzerland). Data are shown as the mean percentage ± SD relative to untreated controls (0%), from five experiments performed in triplicate.

### 2.10. pSRC^Tyr529^/SRC Determination

The phosphorylation status of Src at tyrosine 529 and the total Src protein levels were assessed using cell-based ELISA kits (MyBioSource, San Diego, CA, USA) following the manufacturer’s protocols. Experimental treatments were applied to cells in 96-well plates at the correct density. At the end of the stimulation period, cells were fixed in the provided fixation solution to preserve protein phosphorylation. For the detection of phosphorylated Src (pSrc, Tyr529), cells were incubated with a primary antibody specific to Src phosphorylated at Tyr529, followed by an HRP-conjugated secondary antibody. The substrate was used for colour development, and absorbance was measured at 450 nm using an Infinite 200 Pro MPlex microplate reader (Tecan, Männedorf, Switzerland). In parallel, total Src protein was measured using an ELISA kit that detects all Src isoforms, regardless of phosphorylation status. Absorbance was similarly measured at 450 nm. The relative Src phosphorylation was estimated as pSrc/Src for each condition. Data are shown as mean (%) ± SD, relative to untreated controls (0% line), from five triplicate experiments.

### 2.11. HMGR ELISA Kit

Using the HMGCR ELISA Kit (LSBio, Seattle, DC, USA), HMGR concentrations were measured according to the manufacturer’s instructions [47]. Absorbance at 450 nm was measured using an Infinite 200 Pro MPlex plate reader (Tecan, Männedorf, Switzerland) and calculated from a standard curve ranging from 0.625 to 40 ng/mL. Five separate tests were performed in triplicate, and the results are displayed as mean percentage ± standard deviation, normalised to untreated control cells (set at 0%).

### 2.12. Determination of Total and Free Cholesterol Levels

According to the manufacturer’s instructions [37], a commercial Cholesterol Quantitation Kit (Merck Life Science, Milan, Italy) was used to measure HepG2 cell cholesterol levels. Lipids were extracted from cells using chloroform, isopropanol, and IGEPAL CA-630 (7:11:0.1) and then centrifuged at 13,000× *g* for 10 min after treatment. After evaporating the supernatant at 50 °C, the lipid residue was reconstituted in Cholesterol Assay Buffer with Reaction Mix. Absorbance was measured at 570 nm using an Infinite 200 Pro MPlex microplate reader (Tecan, Männedorf, Switzerland) following 60 min of incubation at 37 °C under light protection. Results are presented as mean percentage ± standard deviation, normalised to protein content (µg/µL), and compared to untreated control cells (0%), from five separate, triplicate experiments.

### 2.13. LDL Uptake Quantification

To measure LDL cholesterol, a colourimetric assay kit (cholesterol oxidase/phenol aminophenazone technique; Elabscience, Wuhan, China) was used [39]. Absorbance was recorded at 546 nm using an Infinite 200 Pro MPlex plate reader (Tecan, Männedorf, Switzerland). Results are shown as mean (%) ± SD, normalised to protein content (µg/µL), and compared to untreated control cells (0% line) from five separate, triplicate experiments.

### 2.14. Bile Acid Production Assay

A fluorometric assay measured total bile acid in HepG2 cells after stimulation [48]. Cells were washed, lysed by sonication in cooled 1× PBS, then centrifuged at 10,000 × *g* for 10 min at 4 °C. Resorufin fluorescence (excitation 560 nm, emission 590 nm) determined bile acid levels using an Infinite 200 Pro MPlex reader. Data are shown as mean percentage ± SD, adjusted to protein content (µg/µL), compared to untreated control cells (0%), from five experiments in triplicate.

### 2.15. SREBP-2 Levels (ELISA Kit)

The manufacturer’s instructions were followed to lyse HepG2 cells and quantify SREBP-2 concentrations with an ELISA kit (LSBio, Seattle, DC, USA) [47]. Absorbance was measured at 450 nm using an Infinite 200 Pro MPlex microplate reader (Tecan, Männedorf, Switzerland). Values were calculated using a standard curve ranging from 0.312 to 20 ng/mL and expressed as ng/µL. Results are shown as the mean percentage ± standard deviation, compared to untreated control cells (0%), from five separate, triplicate experiments.

### 2.16. Western Blot

HepG2 cells were lysed on ice with Complete Tablet Buffer (Roche, Basel, Switzerland) plus 1 mM PMSF, 2 mM Na_3_VO_4_, a 1:50 phosphatase inhibitor cocktail, and a 1:200 protease inhibitor cocktail following standard procedures [49]. Protein samples (35 µg) were separated on 8% SDS-PAGE gels and transferred onto PVDF membranes (GE Healthcare, Chicago, IL, USA). Primary antibodies for LDLr and PCSK9 (1:500; Santa Cruz Biotechnology, Santa Cruz Biotechnology, Dallas, TX, USA) were incubated overnight at 4 °C. Protein loading was validated with anti-β-actin (1:4000; Merck Life Science). Image Lab™ Software (version 5.2.1; Bio-Rad, Hercules, CA, USA) analysed the densitometry and band intensities. Data are shown as mean percentage ± SD relative to control (0%).

### 2.17. Statistical Analysis

All experiments were performed five times with triplicate assays. Western blot data are means ± SD from at least three experiments each performed in triplicate. Data were expressed as percentage-normalised values, with the untreated control defined as 0% to facilitate comparison between treatment groups. Results are presented as mean (%) ± SD. For Western blot experiments, densitometric band intensities were first normalised to the corresponding loading control (β-actin) before calculating percentage variation relative to the untreated control. The percentage deviation from control was calculated using the following formula:

$$\%\;\mathrm{variation}\;=\;(\frac{\;\mathrm{normalized}\;\mathrm{OD}\;\mathrm{treated}}{\;\mathrm{normalized}\;\mathrm{OD}\;\mathrm{control}}-1)\;\times \;100$$

Statistical analyses used GraphPad Prism 10.2.3. Group comparisons employed one-way ANOVA with Bonferroni’s post hoc test; *p* < 0.05 was significant.

## 3. Results

### 3.1. Chemical Analysis of the Extracts

Before biological testing, all extracts were chemically analysed to identify the main bioactive components, and results are expressed as % (*w*/*w*, dry extract; see Table 1). Total polyphenols were measured with the Folin–Ciocalteu assay, while polysaccharides, flavonoids, SAC, and tannins were quantified using targeted colourimetry, acid hydrolysis, or other methods such as HPLC–MS/MS and HPLC-DAD.

The rhizome extract of *Gastrodia elata* exhibited a high polysaccharide content (10.9%) and a polyphenol level of 3.2%, including minor amounts of vanillic acid and gastrodin derivatives, consistent with the literature reports, confirming its potential as a source of neuroprotective compounds. Black Garlic extract contained 0.66% SAC and 5.1% polyphenols (e.g., allixin and allicin derivatives), supporting its antioxidant properties and the presence of cardiovascular-active constituents. *Primula veris* leaf extract (4:1) showed 2.4% polyphenols (mainly caffeic acid derivatives) and 1.2% flavonoids (quercetin and kaempferol derivatives). The hydroethanolic extract of AMLA fruit was further analysed for total polyphenols and tannins, revealing 61.13 ± 3.86 mg gallic acid equivalents (GAE)/g for polyphenols (including gallic acid derivatives) and 32.4 ± 2.1 mg equivalents/g for tannins (emblicanin A/B) correspond to 3.24 ± 0.21% and 6.11 ± 0.39% respectively, confirming a significant proportion of condensed tannins within its phenolic profile.

Overall, these results provide a comprehensive chemical profile of the extracts, establishing a solid basis for correlating their bioactive contents with subsequent biological activities.

### 3.2. Analysis of Dose-Dependent Responses of Botanical Extracts in the Caco-2 Cell Line

**Figure 1:**
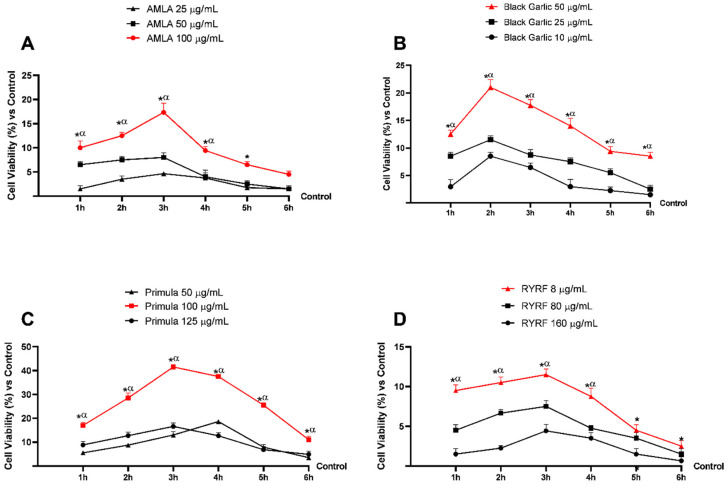
Effects of AMLA, Black Garlic, Primula, and RYRF on Caco-2 cell viability in dose–response and time-course studies (1–6 h). In (**A**), AMLA; in (**B**), Black Garlic; in (**C**), Primula; in (**D**), RYRF viability results. Data are expressed as mean ± SD (%) of five independent experiments, each performed in triplicate (*n* = 5 × 3; biological × technical replicates), and normalised to the untreated control (0%) line. Positive values indicate an increase relative to the untreated control. * *p* < 0.0001 (except for AMLA at 6 h) vs. Control; α *p* < 0.0001 vs. the other concentrations.

The first phase of the study aimed to identify the optimal concentrations of the tested extracts; this step was essential to avoid cytotoxic effects and to determine the concentration that produced the most favourable biological response, as measured by cell viability. Each compound was evaluated at three different concentrations, and Caco-2 cells were treated for time intervals ranging from 1 to 6 h. Cell viability was subsequently assessed at each time point using the MTT assay (Figure 1).

For each sample, the concentration that ensured the highest preservation or enhancement of cell viability compared with both the other tested doses and the untreated control (control; 0% line) was identified. AMLA and Black Garlic (Figure 1A,B) exhibited comparable trends, with the optimal concentration corresponding to the highest dose tested, indicating a dose-dependent effect. Specifically, the most effective non-cytotoxic concentrations were 100 μg/mL for AMLA and 50 μg/mL for Black Garlic (*p* < 0.0001). In contrast, Primula (Figure 1C) displayed a distinct biological profile, characterised by a significant increase in cell viability at an intermediate concentration, followed by a reduction at the highest dose. The optimal concentration for Primula was identified as 100 μg/mL (*p* < 0.0001). Lastly, RYRF (Figure 1D) showed an inverse dose–response relationship, with the most enhancement of cell viability observed at the lowest concentration tested (8 μg/mL) compared with higher doses (*p* < 0.0001).

Based on the results obtained, AMLA 100 μg/mL, Black Garlic 50 μg/mL, and Primula 100 μg/mL were selected for subsequent experimental phases and combined with *Gastrodia elata* 100 μg/mL. The combination of these four extracts is hereafter referred to as the MIX and was evaluated against the individual extracts and RYRF at 8 μg/mL. An LC–MS/MS analysis of the MIX confirmed it maintained expected levels of each bioactive compound, reflecting the contributions of individual extracts, with no notable quantitative differences after mixing (see Table A1 in Appendix A).

### 3.3. Assessment of Intestinal-Barrier Function Following Treatment with the Botanical Combination

**Figure 2:**
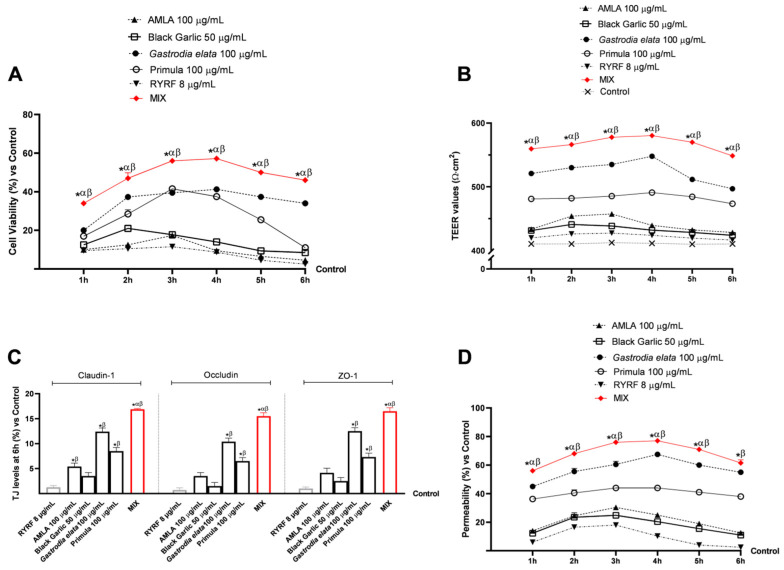
Results of treatments over the 1–6 h period in an in vitro 3D intestinal barrier model. In (**A**), cell viability; in (**B**), TEER measurements; in (**C**), tight junction analysis (claudin-1, occludin, and ZO-1 proteins levels); and in (**D**), permeability detection. Data are presented as mean ± SD (%) from five independent experiments, each performed in triplicate (*n* = 5 × 3; biological × technical replicates), normalised to the untreated control (0% line only in A, C, D). Positive values indicate an increase relative to the untreated control. * *p* < 0.0001 vs. Control; α *p* < 0.0001 vs. single botanical extract; β *p* < 0.0001 vs. RYRF 8 μg/mL.

During the second analytical phase, data were collected on the safety, preservation of integrity, and functionality of the in vitro intestinal-barrier model cultured on Transwell inserts following treatment with the test samples (Figure 2). All evaluations were performed within a treatment time window ranging from 1 to 6 h. Initially, positive effects on cell viability were observed (Figure 2A), confirming previous findings for the individual compounds and showing a statistically significant increase for the MIX. In particular, the MIX showed a statistically significant increase in cell viability compared with all individual agents across the entire time course analysed (*p* < 0.0001), reaching a maximum at 4 h (57% vs. control; *p* < 0.0001).

Consistent results were also obtained in the evaluation of intestinal-barrier integrity as assessed by TEER measurements (Figure 2B). All tested samples maintained TEER values above the established cut-off (400 Ω·cm^2^). Each individual extract exhibited higher TEER values compared with RYRF at 8 μg/mL, with *Gastrodia elata* at 100 μg/mL showing the highest TEER values among single compounds. Notably, the MIX produced significantly greater TEER values than both the individual extracts and RYRF (8 μg/mL), reaching peak levels between 3 and 4 h of treatment (580 Ω·cm^2^).

In line with TEER measurements, the 6 h analysis of tight junction (TJ) proteins (Figure 2C) indicated that all extracts led to an increase in Claudin-4, Occludin, and ZO-1 expression compared to the control. The highest levels were observed with *Gastrodia elata* at 100 μg/mL and Primula at 100 μg/mL. The MIX showed a moderate additional effect, reaching 15–17% and exhibiting a statistically significant difference compared to the individual components and RYRF at 8 μg/mL (*p* < 0.0014).

Finally, as shown in Figure 2D, reliable data were obtained regarding permeability across the in vitro intestinal barrier model over the 1–6 h treatment period. Consistent with previous findings, all individual extracts exhibited higher permeability values than RYRF at 8 μg/mL, with *Gastrodia elata* at 100 μg/mL showing significantly superior permeability, exceeding 40% throughout the entire observation period. In contrast, AMLA 100 μg/mL, Black Garlic 50 μg/mL, and Primula 100 μg/mL exhibited comparable permeability profiles (12–35%), with maximal transport around 3 h. MIX led to higher permeability than any individual extract (*p* < 0.0001). The recorded data were found to be above 50% for all studied time frames, with a peak absorption of 77% at 4 h. Around the peak phase, MIX increased permeability by 61% compared to AMLA 100 μg/mL, by 68% compared to Black Garlic 50 μg/mL, by 17% compared to *Gastrodia elata* 100 μg/mL and by 43% compared to Primula 100 μg/mL, with data 75% higher than that of RYRF 8 μg/mL.

### 3.4. Effects of Botanical Combination on Hepatic Cell Function and Signalling After Intestinal Absorption

**Figure 3:**
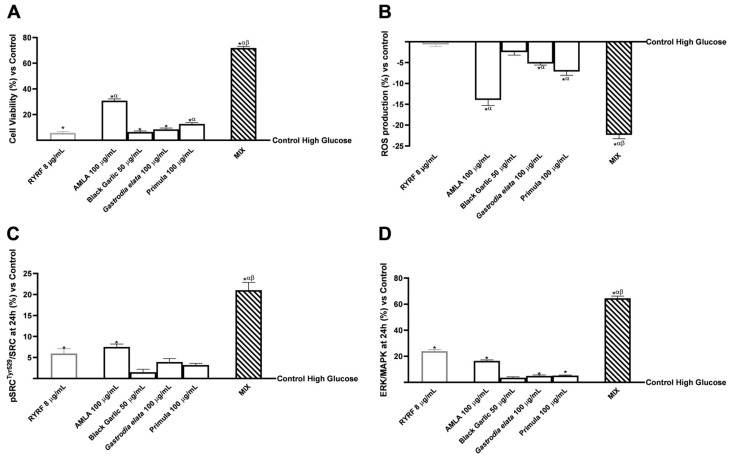
Effects of sample treatments over a 24 h period on HepG2 cell viability, oxidative stress, and modulation of SRC and MAPK signalling pathways following intestinal absorption. In (**A**), cell viability; in (**B**), ROS production results obtained; in (**C**), pSRC^Tyr529^/SRC ratio and in (**D**) ERK/MAPK ratio. Line 0% is replaced with untreated cells (untreated control) under high glucose conditions (D-glucose 30 mM). The data is shown as mean ± SD (%) from five separate experiments, each performed in triplicate (*n* = 5 × 3; biological × technical replicates), normalised to the untreated control (line 0%). Positive values indicate an increase, whereas negative values indicate a reduction relative to the untreated control. * *p* < 0.05 vs. Control; α *p* < 0.0001 vs. RYRF 8 μg/mL; β *p* < 0.0001 vs. single botanical extract (except for ROS production in which *p* < 0.0003).

In this part of the study, the biological effects of combining four botanical extracts with RYRF at 8 μg/mL on HepG2 cells following intestinal passage were evaluated (Figure 3). Specifically, HepG2 cells were treated for 24 h with supernatants collected from the intestinal-barrier model after exposure to each test sample. The safety profile was first assessed through cell viability (MTT assay, Figure 3A) and oxidative stress (cytochrome C colourimetric assay, Figure 3B). In addition, the activation of SRC and MAPK pathways was assessed by measuring the pSRC^Tyr529^/SRC and ERK/MAPK ratios (Figure 3C,D).

To mimic hypercholesterolemic conditions in vitro, HepG2 cells were exposed to high glucose levels (30 mM D-glucose) [42]. All experimental data were compared to untreated cells maintained under the same high-glucose conditions (High Glucose Control; 0% baseline in the graphs).

Figure 3A shows that all extracts significantly maintained or increased cell viability compared to the control (*p* < 0.05). Effects comparable to the reference sample RYRF 8 μg/mL were observed with Black Garlic 50 μg/mL and *Gastrodia elata* 100 μg/mL. In contrast, Primula 100 μg/mL and especially AMLA 100 μg/mL produced significantly higher effects than RYRF (8 μg/mL) (*p* < 0.05). Notably, MIX elicited an increase compared with either the individual extracts or RYRF 8 μg/mL (*p* < 0.0001), preliminarily indicating a potential synergistic interaction that exceeds the additive effects of the single components. To complement a post hoc analysis of Bliss independence, this is presented in Appendix A (Table A2), which indicates that the observed combined effects were greater than the expected additive responses. Regarding oxidative stress (Figure 3B), all tested samples reduced ROS production relative to the untreated control, with statistical significance in all cases except RYRF at 8 μg/mL (*p* < 0.05). Notably, each individual botanical extract exhibited a stronger antioxidant effect than RYRF alone, with statistically significant differences (*p* < 0.05), except for Black Garlic 50 μg/mL. Moreover, the MIX further enhanced the protective effects observed for the single extracts, showing a further reduction in ROS when the extracts were combined (*p* < 0.0003).

Consistent with the observed cytoprotective and antioxidant effects, analysis of intracellular signalling pathways highlighted a modulation of the SRC and ERK/MAPK pathways (Figure 3C,D). An increase in the pSRC^Tyr529^/SRC ratio was observed, indicative of a reduction in SRC levels, together with an increase in the ERK/MAPK ratio, associated with pro-survival mechanisms across all samples examined, though not always statistically significant. Among the individual agents, AMLA 100 μg/mL outlined the best bioactive effects in both markers investigated and, in some cases, (pSRC^Tyr529^/SRC), higher than RYRF 8 μg/mL. MIX also led to increases in these intracellular markers compared with the individual extracts (*p* < 0.0001). In detail, the MIX increased the pSRC^Tyr529^/SRC ratio by 58% compared to AMLA 100 μg/mL, by 91% compared to Black Garlic 50 μg/mL, by 88% compared to *Gastrodia elata* at 100 μg/mL and by 83% compared to Primula 100 μg/mL, with data 92% higher than also RYRF 8 μg/mL. At the same time, MIX increased the ERK/MAPK ratio by 85% compared to AMLA 100 μg/mL, by 96% compared to the other botanical extracts, with data 80% higher than RYRF 8 μg/mL.

### 3.5. Biological Activity of the Botanical Extract Combination on Cholesterol Metabolism

**Figure 4:**
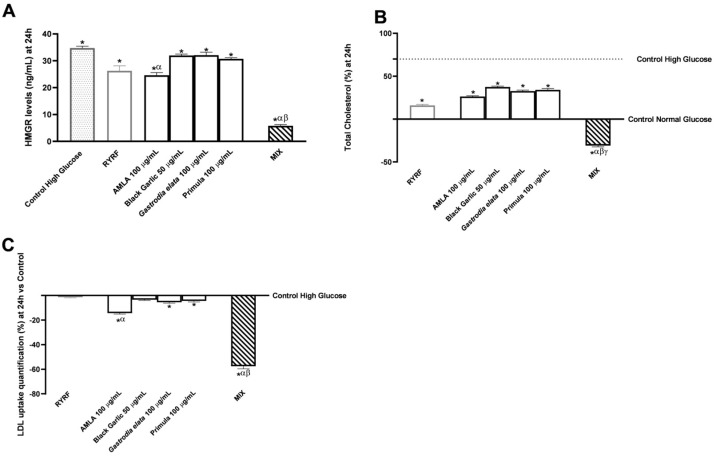
Modulation of cholesterol homeostasis by a botanical extract combination in HepG2 Cells under high-glucose conditions. In (**A**) HMGR protein levels (ng/mL). In (**B**) Total cholesterol levels. In (**C**), LDL uptake. The 0% line (in (**B**) represented as a dotted line at 70%) corresponds to untreated cells cultured under high-glucose conditions (D-glucose 30 mM). In (**C**), the dotted line shows high glucose control (30 mM), and the 0% line indicates normal glucose control (5 mM). Data are presented as mean ± SD (%) from five independent experiments, each performed in triplicate (*n* = 5 × 3; biological × technical replicates), and normalised to the untreated control. In (**B**,**C**), positive values indicate an increase, whereas negative values indicate a reduction relative to untreated controls. * *p* < 0.05 vs. Control; α *p* < 0.0001 vs. RYRF; β *p* < 0.0001 vs. single botanical extract; γ *p* < 0.0001 vs. Control Normal Glucose.

HepG2 cells were cultured in high-glucose medium to mimic hyperglycaemic stress. Figure 4 illustrates how botanical extracts and their combination influenced HMGR, total cholesterol, and LDL uptake in the liver (Figure 4).

Figure 4A, represented as a reduction in HMGR ng/mL levels, shows that after 24 h of treatment, each individual botanical extract, as well as RYRF at 8 μg/mL, significantly reduced HMGR levels compared with the untreated control (*p* < 0.05). Among the individual treatments, AMLA 100 μg/mL demonstrated one of the more pronounced inhibitory effects on HMGR, with significant data compared to RYRF 8 μg/mL. MIX significantly reduced HMGR levels more than individual extracts or RYRF at 8 μg/mL (*p* < 0.0001), indicating effective suppression of cholesterol synthesis and potential synergy. Appendix A (Table A3) shows that actual combined effects exceeded predicted additive responses per Bliss independence. Specifically, treatment with the combined formulation lowered HMGR levels by 83.2% compared with the untreated control, by 66% compared to AMLA 100 μg/mL, by 90% compared to Black Garlic 50 μg/mL and *Gastrodia elata* 100 μg/mL and by 86% compared to Primula 100 μg/mL, with data 78% higher than also RYRF 8 μg/mL.

Figure 4B shows that treatments modestly but significantly lowered cholesterol in HepG2 cells compared to high-glucose control (*p* < 0.05). Among the single treatments, AMLA 100 μg/mL showed the most pronounced effect compared with the high-glucose control. MIX, however, produced a much stronger decrease, demonstrating superior ability to limit cholesterol accumulation. Compared with the untreated control, total cholesterol was reduced by 8-fold, 1.9-fold compared with AMLA 100 μg/mL, 4-fold compared with the other single extracts, and 1.8-fold relative to RYRF 8 μg/mL.

Analysis of LDL uptake further supported the modulation of cholesterol metabolism (Figure 4C). While the reductions induced by individual botanical extracts were not always statistically significant compared with RYRF 8 μg/mL (except for AMLA 100 μg/mL, *p* < 0.0001), all extracts showed a decrease in LDL uptake compared to the untreated control (*p* < 0.05 except for Black Garlic 50 μg/mL) and RYRF 8 μg/mL. AMLA 100 μg/mL produced a statistically significant reduction versus the untreated control (*p* < 0.0001). Notably, MIX significantly enhanced the impact of the individual extracts on LDL uptake (*p* < 0.0001), reducing it by 36.7% relative to the untreated control, by 75% compared with AMLA 100 μg/mL, by 88% compared with the other single extracts, and by 98% versus RYRF 8 μg/mL. Appendix A (Table A4) supplements the post hoc analysis using Bliss independence, showing that the observed combined effects exceeded the predicted additive responses. Raw data related to the results displayed in Figure 4 are provided in Appendix B (Table A8, Table A9 and Table A10).

**Figure 5:**
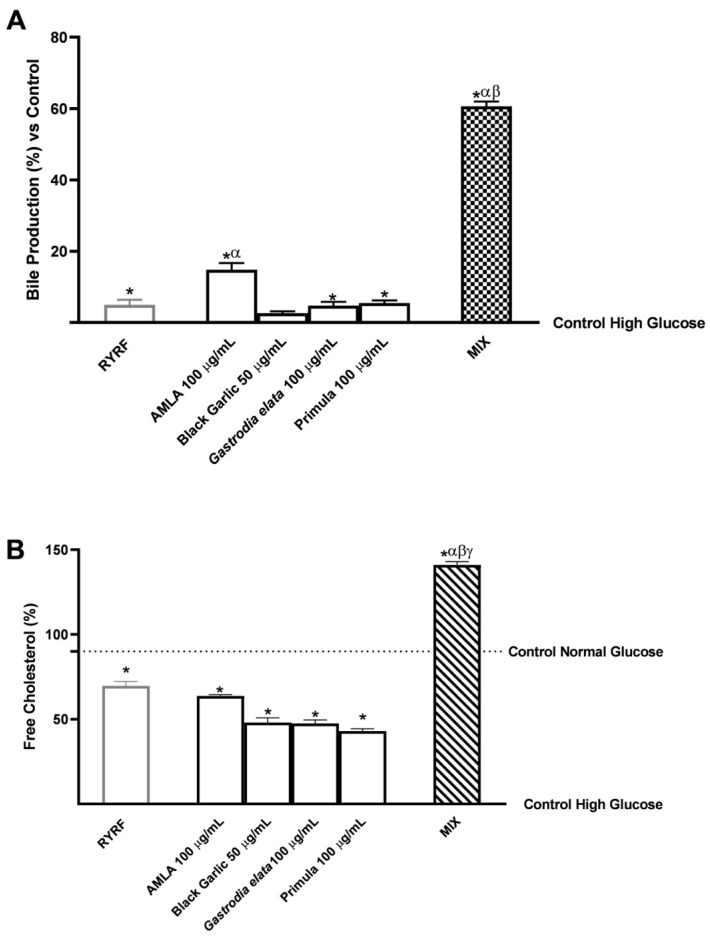
Impact of the botanical extract mixture after 24 h treatment on cholesterol elimination pathways in HepG2 cells in high-glucose conditions. In (**A**), Bile production. In (**B**), free cholesterol content. The 0% reference line (with the exception of (**B**)) corresponds to untreated cells cultured under high-glucose conditions (30 mM D-glucose). The dotted line represents normal-glucose control. Results are expressed as mean ± SD (%) from five independent experiments, each performed in triplicate (*n* = 5 × 3; biological × technical replicates), and are normalised to the untreated high-glucose control (0% line, except for Figure 3B). Positive values indicate an increase relative to untreated controls. * *p* < 0.05 vs. Control; α *p* < 0.0001 (except for AMLA in bile production, *p* < 0.0008) vs. RYRF; β *p* < 0.0001 vs. single botanical extract; γ *p* < 0.0001 vs. normal-glucose control.

Additional investigations focused on major determinants of cholesterol elimination, considered within the framework of hepatic cholesterol metabolism and homeostasis. Specifically, bile acid production and free cholesterol release were evaluated (Figure 5).

As shown in Figure 5A, every treatment significantly enhanced bile acid production relative to the untreated control (*p* < 0.05 except for Black Garlic 50 μg/mL). In contrast to *Gastrodia elata* and Primula at 100 μg/mL, which showed bile production levels comparable to those observed with RYRF at 8 μg/mL, AMLA 100 μg/mL elicited a relatively higher response, resulting in higher bile production than RYRF at 8 μg/mL (*p* < 0.0008). MIX led to a further increase in data from the individual botanical constituents, with statistically significant results compared to RYRF 8 μg/mL alone (*p* < 0.0001). Quantitatively, MIX increased bile production by an average of 61% compared with the untreated control, by 75% compared with AMLA 100 μg/mL, and by approximately 93% compared with the other botanical components and RYRF at 8 μg/mL. As shown in Appendix A (Table A5), the post hoc analysis using Bliss independence reveals that the observed combined effects surpass the predicted additive outcomes. Raw data corresponding to the results shown in Figure 5A are provided in Appendix B (Table A11).

Figure 5B reports the corresponding data for excreted free cholesterol, with values also compared to those measured in the normal-glucose control condition (indicated by the dotted line). Exposure to the individual botanical extracts or RYRF 8 μg/mL significantly elevated free cholesterol levels compared with the high-glucose control (*p* < 0.0001); however, these levels did not reach those observed under normal-glucose conditions. AMLA 100 μg/mL showed effects comparable to those of RYRF 8 μg/mL alone. MIX displayed a distinct biological effect, producing a significant increase in free cholesterol relative to the high-glucose control (*p* < 0.0001) and reaching levels above the normal-glucose control (*p* < 0.0001). Moreover, this increase was significantly greater than that achieved with any single component (*p* < 0.0001). On average, the nutraceutical formulation increased free cholesterol by 1.41-fold compared with the untreated high-glucose control, by 36% relative to the untreated normal-glucose control, by 53% compared with AMLA 100 μg/mL, by 64% compared with the other botanical extracts, and by 50% compared with RYRF 8 μg/mL.

### 3.6. Modulation of the PCSK9–LDLr–SREBP-2 Signalling Network by the Botanical Extract Blend

**Figure 6:**
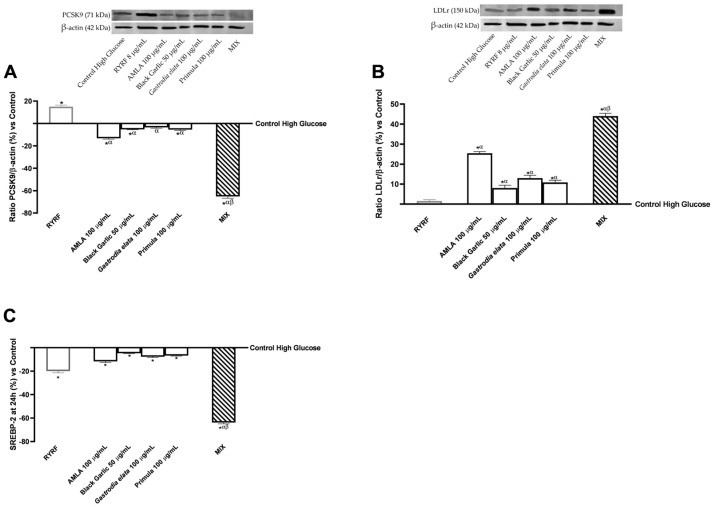
Effects of the nutraceutical extract combination after 24 h treatment on PCSK9–LDLR–SREBP-2 regulatory Axis in HepG2 cells under high-glucose conditions. In (**A**) PCSK9, (**B**) LDLR densitometric analysis with an example image for each. In (**C**), SREBP-2 levels. The line 0% is remade to untreated cells (untreated control) under high glucose (D-glucose 30 mM) conditions. The ELISA data are shown as mean ± SD (%) from five separate experiments, each performed in triplicate *(n* = 5 × 3; biological × technical replicates), normalised to the untreated control (line 0%). Western blot data are presented as mean ± SD (%) from three independent experiments, each performed in triplicate (*n* = 3 × 3; biological × technical replicates), normalised to the untreated control. Positive values indicate an increase, whereas negative values indicate a reduction relative to the untreated control. * *p* < 0.0001 vs. Control; α *p* < 0.0001 vs. RYRF (except for single extracts in LDLR expression, *p* < 0.008); β *p* < 0.0001 vs. single botanical extract.

Using a high-glucose HepG2 model to induce metabolic stress, further analyses investigated the test samples’ effects on the PCSK9–LDLr–SREBP-2 axis after 24 h (Figure 6).

In the PCSK9 analysis (Figure 6A), densitometric quantification of Western blot signals revealed that treatment with RYRF 8 μg/mL led to a significant upregulation of PCSK9 protein levels relative to the untreated high-glucose control (*p* < 0.0001). In contrast, each botanical extract tested individually produced a significant reduction in PCSK9 expression compared with RYRF at 8 μg/mL (*p* < 0.0001). These decreases were also significant when compared with the untreated high-glucose control (*p* < 0.0001), except for Black Garlic 50 μg/mL. Among the single treatments, AMLA 100 μg/mL showed the most pronounced effect. Consistent with previous observations, MIX induced a further reduction in PCSK9 protein expression, as determined by Western blot densitometry, with levels significantly lower than those obtained with any single constituent (*p* < 0.0001). Specifically, the MIX reduced PCSK9 expression by 80% relative to AMLA at 100 μg/mL, by 92% relative to the other individual botanical extracts, and by 5-fold relative to RYRF at 8 μg/mL. Appendix A (Table A6) shows Bliss independence analysis indicates combined effects surpass expected additivity; raw data for Figure 6A are in Appendix B (Table A12).

Accordingly, LDLR expression assessed by Western blot analysis (Figure 6B) indicated that the botanical substances under investigation exhibited relatively higher response. Compared with the high-glucose control and RYRF at 8 μg/mL, single extracts induced a progressive increase in LDLR expression, with quantitative differences among treatments and statistically significant effects versus both RYRF 8 μg/mL and untreated control (*p* < 0.008). After 24 h, AMLA 100 μg/mL produced the most notable effects among individual extracts. The MIX treatment elicited the highest and statistically significant increase in LDLR expression (*p* < 0.0001), with increases of 43% versus AMLA 100 μg/mL, 81% versus Black Garlic 50 μg/mL, 70% versus *Gastrodia elata* 100 μg/mL, 75% versus Primula 100 μg/mL, and 97% versus RYRF 8 μg/mL.

The SREBP-2 analysis (Figure 6C) showed a similar pattern among the botanical extracts and RYRF at 8 μg/mL, both of which significantly reduced SREBP-2 levels compared to the untreated high-glucose control (*p* < 0.0001). Individual botanical extracts produced weaker effects than RYRF 8 μg/mL alone. In contrast, the MIX treatment led to a further reduction in SREBP-2 levels compared with the individual extracts and RYRF 8 μg/mL. Specifically, MIX reduced SREBP-2 by 81% versus AMLA 100 μg/mL, by 89% versus the other individual botanical extracts, and by 68% versus RYRF 8 μg/mL. Appendix A (Table A7) complements the post hoc Bliss independence analysis by demonstrating that the observed combined effects are greater than those predicted by additivity.

## 4. Discussion

The gut–liver axis is a central regulator of lipid metabolism, linking intestinal nutrient handling, microbial metabolites, and hepatic metabolic responses [4]. Intestinal barrier breakdowns allow endotoxins such as lipopolysaccharide to enter the portal circulation, causing hepatic inflammation, dyslipidaemia, and NAFLD [50]. These mechanisms are increasingly recognised as contributors to hypercholesterolemia and cardiometabolic risk beyond traditional lipid synthesis pathways. Pharmacological therapies (e.g., statins) remain the mainstay for LDL-cholesterol reduction, acting primarily on hepatic HMGR, yet they may not address gut-derived inflammatory influences [51,52]. Nutraceuticals, especially combinations of bioactive plant extracts, have been investigated for complementary modulation of lipid metabolism through integrated gut and liver effects, including impacts on bile acid signalling and microbial composition [53,54]. Consistently, our preliminary chemical analyses showed that each extract possesses a distinct bioactive profile: *Gastrodia elata* is rich in polysaccharides, Black Garlic contains good levels of SAC and polyphenols, Primula leaves provide moderate polyphenols and flavonoids, and AMLA fruit offers substantial polyphenols and condensed tannins. These differences likely contribute to their complementary antioxidants and lipid-modulating potentials, supporting the rationale for their combined use in hypothetical oral nutraceutical formulations.

In the context of oral supplementation, the integrity of the intestinal epithelial barrier is fundamental for the controlled absorption of nutrients and other compounds, preventing excessive paracellular leakage that leads to metabolic endotoxemia and liver stress [55,56]. The Caco-2 cell monolayer is a validated in vitro model of the human small intestinal epithelium. Upon differentiation, these cells display enterocyte-like features, form tight junctions, and exhibit polarised transport, providing a useful system for studying intestinal absorption and related processes [57,58,59]. Our results indicate that the MIX not only preserved cell viability but also enhanced TEER, indicating enhanced tight junction integrity and modulated paracellular permeability [60] compared with untreated controls and single extracts. This is consistent with evidence from the literature that optimised formulations significantly enhance intestinal permeability and the transport of complex bioactives [60,61]. Increased TEER does not necessarily limit overall transport of bioactive compounds, as their passage can occur via transcellular pathways independent of tight junctions [62,63]. Therefore, the observed enhancement in TEER can coexist with efficient absorption of complex bioactives, reflecting both strengthened barrier function and preserved transcellular uptake mechanisms. The permeability values observed for MIX in the Caco-2 model (>70%) are consistent with well-absorbed compounds in vitro, provided barrier integrity is maintained. Compounds with Papp > 10 × 10^−6^ cm/s generally correspond to high intestinal absorption (70–100%) via both passive transcellular and paracellular pathways, in line with Biopharmaceutics Classification System (BCS) guidelines for predicting oral uptake [64,65]. The significantly increased permeability observed with MIX may reflect additive or synergistic effects on epithelial transport mechanisms, potentially involving modulation of TJ dynamics, as indicated by the observed increased expression of claudin-4, occludin, and ZO-1 [66]. While the precise mechanisms remain to be elucidated, these findings suggest that multicomponent nutraceuticals can enhance intestinal uptake of multiple actives, consistent with preclinical studies showing greater effects for combinations than single compounds. The maintained barrier integrity indicates that the permeability increase occurs within physiological limits rather than from epithelial disruption [67,68].

Following the intestinal absorption and passage phase, HepG2 cells cultured under high-glucose conditions represent a widely accepted in vitro model that reproduces key aspects of insulin resistance and dysregulated lipid metabolism [36,69,70]. First, the samples’ liver safety under high-glucose conditions is demonstrated both in terms of viability and oxidative stress. This last aspect is evident in the MIX of botanical extracts due to their bioactive content and the hypothetical combined antioxidant action [30,31]. Furthermore, the extracts were associated with modulation of SRC and ERK/MAPK pathways, as indicated by increases in the pSRC^Tyr529^/SRC and ERK/MAPK ratios across all treatments, reflecting correlative activation of pro-survival signalling [71,72]. These findings are consistent with the literature, where the SRC and ERK/MAPK pathways have been linked to oxidative stress responses, cytoprotection, and regulation of apoptosis and autophagy in hepatocytes, as well as in antifibrotic and metabolic processes [73,74]. The findings do not establish direct mechanistic causality but support an associative network in which modulation of oxidative stress can influence these interconnected kinases and downstream cytoprotective pathways.

Numerous studies have validated the high-glucose condition model in HepG2 cells for assessing the modulation of hepatic lipid metabolism by nutraceuticals and phytochemicals, with strong translational relevance to in vivo conditions [75,76]. This in vitro system enables the integrated evaluation of central regulators of cholesterol homeostasis, including HMGR, LDLR, PCSK9, and the transcription factor SREBP-2, which collectively coordinate cholesterol synthesis, uptake, and feedback control mechanisms [14]. In this phase, the inclusion of RYRF as a reference was fundamental because it contains monacolin K, a compound structurally and functionally related to statins, widely used as a standard for comparison in cholesterol-lowering interventions [77]. Thus, RYRF serves as a pharmacological benchmark, allowing the relative evaluation of the efficacy of individual extracts and their combination in modulating hepatic lipid metabolism. Importantly, the botanical extracts were also assessed to preliminarily outline mechanisms of action different from the statin-mimetic effects typical of monacolin K [8].

HMGR, the rate-limiting enzyme in de novo cholesterol biosynthesis, is a primary molecular target for lipid-lowering strategies. Several botanical extracts rich in polyphenols, organic acids, and sulfur-containing compounds [78,79], such as those present in AMLA (gallic acid, ellagic acid), Black Garlic (SAC), have been shown to downregulate HMGR expression or activity in HepG2 cells and animal models [79,80,81]. Importantly, combinations of phytochemicals have demonstrated superior inhibitory effects on HMGR expression compared with single compounds, as reported for synergistic mixtures including chlorogenic acid, quercetin, and iridoid glycosides [82]. In line with these observations, the pronounced suppression of HMGR observed with the MIX in our study supports the hypothesis that chemically complementary bioactives may converge on cholesterol biosynthesis pathways more effectively than isolated extracts or RYRF.

Beyond regulating cholesterol synthesis and uptake, hepatic cholesterol elimination via bile acid production represents an essential mechanism for maintaining cholesterol homeostasis. Bile acids are synthesised from cholesterol primarily via the rate-limiting enzyme CYP7A1 in the classic pathway, and enhanced formation facilitates cholesterol elimination and excretion. Polyphenols, flavonoids and simple phenolics (such as Quercetin and kaempferol, also contained in *Gastrodia elata* and *Primula veris*, have been shown in animal and in vitro studies to modulate CYP7A1 expression and increase bile acid synthesis and excretion, thereby contributing to reductions in total and LDL cholesterol [19,22]. In addition, AMLA and Black Garlic have been shown, in vitro and in vivo, to improve cholesterol metabolism and lipid turnover, with effects on lipid profiles and on pathways influencing bile acid homeostasis [15,83,84]. In our study, the MIX significantly increased bile acid production compared with single extracts and RYRF, indicating that the combinatorial approach not only suppresses cholesterol synthesis and enhances LDLR-mediated clearance but also stimulates cholesterol removal through bile formation. This integrative effect reinforces the therapeutic potential of multi-ingredient nutraceuticals in modulating hepatic cholesterol homeostasis and managing hypercholesterolemic conditions.

The LDLR-mediated uptake of circulating LDL particles is a central mechanism for hepatic cholesterol clearance, tightly regulated by PCSK9, which binds LDLR and directs it to lysosomal degradation, and by transcription factors such as SREBP-2 and HNF1α [85]. Combining botanical extracts rich in polyphenols, alkaloids and sulfur-containing compounds has allowed in vitro modulation of this pathway, enhancing LDLR expression and reducing PCSK9 levels, thereby improving hepatic LDL clearance. AMLA extract, rich in gallic acid, can increase LDLR and decrease PCSK9 expression in HepG2 cells and hyperlipidemic rodents, in part by suppressing hepatocyte nuclear factor 1 Alpha (HNF1α) and modulating SREBP-2, thereby improving LDL uptake and reducing plasma cholesterol [16,86]. Black Garlic, via organosulfur compounds like allicin, upregulates LDLR and downregulates PCSK9 in HepG2 cells through adenosine monophosphate-activated protein kinase (AMPK)/SREBP-2 and ERK1/2 pathways, and improves hepatic lipid metabolism in high-fat diet rodent models [17]. *Gastrodia elata*, containing gastrodin, reduces PCSK9 and enhances LDLR expression in vivo by modulating HNF1α, activating forkhead box protein O3 (FoxO3a), and inhibiting Janus kinase 2/signal transducer and activator of transcription 3 (JAK2/STAT3) signalling, thereby improving LDL-C clearance and decreasing hepatic cholesterol accumulation [19]. Finally, *Primula veris* may, regarding its bioactive content, contribute to increased LDLR and reduced PCSK9 in hepatic cells via SREBP-2 modulation, as observed in the literature and in animal studies, thereby improving hepatic lipid profiles [87,88], although direct PCSK9 effects are less documented.

The in vitro study demonstrates that a botanical formulation containing multiple extracts can regulate hepatic cholesterol balance through multifactorial, synergistic mechanisms. The extracts jointly influence LDLR synthesis, PCSK9-mediated degradation, and upstream transcriptional pathways, enhancing LDL uptake in hepatocytes and promoting cholesterol balance in vivo. These activities are distinct from monacolin-containing RYRF, which primarily inhibits HMGR. The study also provides fresh information about less-studied components, such as *Gastrodia elata* and *Primula veris*, and employs an in vitro gut–liver axis model to simulate nutrient–hepatic metabolic interactions. Although RYRF serves as a useful reference, its lipid-lowering effect is primarily mediated by direct HMG-CoA reductase inhibition via monacolin K, whereas the botanical formulation appears to act through coordinated modulation of HMGR expression, PCSK9, and LDLR. These mechanistic differences suggest that the observed effects of the formulation reflect a broader regulatory profile rather than a statin-like mode of action.

This study relies on a preclinical, in vitro gut–liver axis model, which represents an innovative approach to simultaneously assess intestinal absorption, barrier integrity, and hepatic metabolism in a controlled setting. The model allowed us to investigate the specific combination of MIX and their potential synergistic effects, providing mechanistic insights that would be difficult to capture in simpler single-tissue systems. Indeed, Caco-2/HepG2 coupled system enables investigation of intestinal absorption and hepatic metabolism in vitro, providing a controlled, mechanistic platform to study gut–liver interactions and the effects of botanical formulation [89]. In addition, results on the combined anti-hypercholesterolemic effects in vitro are consistent with evidence that multi-botanical formulations (e.g., AMLA, Olive, Walnut) can exert additive or synergistic lipid-lowering activity not only in HepG2 cells but also in humans [90,91]. Similarly, polyherbal formulations including Garlic and other botanicals decreased LDL and triglycerides and improved antioxidant capacity of single extracts in high-fat-diet-fed rats and in vitro assays [92,93]. These findings suggest that mixtures of botanicals can enhance lipid metabolism beyond the effects of single extracts, although direct studies combining AMLA, Black Garlic, *Gastrodia elata* and *Primula veris*, and remain scarce, highlighting the novelty of the present MIX approach.

However, despite these advantages, several limitations should be highlighted. The first limitation of this study is the semi-quantitative nature of phytochemical characterisation. While major compounds were identified and quantified, full metabolomic profiling was not achieved. Therefore, mechanistic links between specific constituents and the observed effects remain preliminary. Future studies using advanced techniques, such as targeted and untargeted LC-MS/MS for polyphenols and other metabolites, are needed to clarify the underlying molecular mechanisms.

Secondly, the model cannot fully replicate the complexity of human physiology, including systemic interactions, immune responses, and long-term adaptive mechanisms. While increased permeability may suggest enhanced transport potential, subtle changes in barrier properties cannot be excluded, highlighting the need for careful interpretation and further in vivo validation. At the same time, elevated glucose levels induce a hypercholesterolemia-like phenotype through metabolic stress; they do not fully mimic lipid overload, and the in vitro model condition used captures key aspects of glucose-induced hepatic lipid stress, but it may have limited relevance for primary genetic hypercholesterolemia.

The study was conducted over a relatively short period, limiting the ability to evaluate chronic effects or compensatory changes. Moreover, although a lipid-specific challenge (e.g., LDL, oxLDL, or palmitate) would be ideal for mechanistic insights, this was beyond the scope of the current study and is suggested as a potential follow-up. The intrinsic variability of natural extracts may influence the reproducibility and consistency of results. The concentrations used (50–100 μg/mL for botanical extracts and 8 μg/mL for RYRF) were selected based on dose–response and cytotoxicity testing to ensure non-toxic biological activity. These values represent total extract amounts rather than equivalent levels of specific actives. RYRF, standardised for monacolins such as monacolin K, acts as a direct HMGR inhibitor at low concentrations, whereas botanical extracts are complex matrices requiring higher in vitro doses to elicit measurable multi-target effects. In vitro concentrations cannot be directly extrapolated to human plasma levels, as cell models do not reflect metabolism or tissue distribution. Therefore, the tested doses should be considered mechanistic ranges rather than indicators of clinical equivalence.

Taken together, these results highlight promising mechanistic and functional effects, providing significant preclinical information on the biological potential of this nutraceutical strategy and serving as a proof-of-concept for multi-target modulation along the gut–liver axis. Although not indicative of clinical dose equivalence, they offer a solid rationale for further in vivo and clinical validation. The lack of gut microbiota prevents the complete recapitulation of gut–liver axis physiology, but the current in vitro model can contribute in the form of a simplified and controlled system to preliminarily analyse epithelial–hepatic communication and support hypothesis-driven translational research. A rigorous pharmacological classification of synergy would require complete dose–response matrices and formal combination index or isobologram analyses; therefore, conclusions regarding synergism should be interpreted with caution.

## 5. Conclusions

This study shows that a multi-extract botanical formulation supports liver cholesterol balance by regulating LDLR, PCSK9, and transcription pathways. The formulation enhanced LDL uptake in hepatocytes via mechanisms distinct from monacolin-containing red yeast rice, while less-studied components such as *Primula veris* may contribute additional roles in lipid metabolism. The in vitro gut–liver axis model, although limited by the absence of gut microbiota and short exposure times, proved valuable for exploring nutrient–metabolism interactions and identifying potential mechanistic targets. These preclinical findings support the translational potential of the formulation and justify further in vivo and clinical studies to confirm efficacy, safety, and long-term cardiometabolic benefits.

## Figures and Tables

**Table 1:** The levels of polyphenols, flavonoids, and polysaccharides in *Gastrodia elata*, Black Garlic extract, *Primula veris*, and AMLA extract were measured using colourimetric techniques, while SAC in Black Garlic extract was quantified by HPLC–MS/MS and tannins in AMLA extract by HPLC-DAD using gallic acid as a reference standard. Data are presented as mean % (*w*/*w*, dry extract) ± SD (*n* = 3). Content values expressed in % *w*/*w* refer to the weight of the compound relative to the dry extract powder. Discrepancies in bioactive component content among the four extracts are shown in the table, guiding biological assessments.

Natural Extract	Methods	Main Bioactive Compounds	Content
*Gastrodia elata*	UV-Vis	Polysaccharides (glucans, galactomannans)	10.9 ± 0.3% *w*/*w* dry extract
	UV-Vis	Polyphenols (vanillic acid, gastrodin derivatives)	3.2 ± 0.1% *w*/*w* dry extract
*Black Garlic*	HPLC	SAC	0.66 ± 0.02% *w*/*w* dry extract
	UV-Vis	Polyphenols (allixin, allicin derivatives)	5.1 ± 0.2% *w*/*w* dry extract
*Primula veris*	UV-Vis	Polyphenols (quercetin, kaempferol derivatives)	2.4 ± 0.1% *w*/*w* dry extract
	UV-Vis	Flavonoids (caffeic acid derivatives)	1.2 ± 0.05% *w*/*w* dry extract
*AMLA*	HPLC	Tannins (emblicanin A/B, punigluconin)	3.24 ± 0.21% *w*/*w* dry extract
	UV-Vis	Polyphenol (gallic acid derivatives)	6.11 ± 0.39% *w*/*w* dry extract

## Data Availability

Data are available from the corresponding author upon reasonable request and for justified scientific reasons, since they relate to a patented substance.

## Abbreviations

The following abbreviations are used in this manuscript:

AMPK	Adenosine Monophosphate-activated Protein Kinase
ATCC	American Type Culture Collection
DMEM	Dulbecco’s Modified Eagle’s Medium
DMEM-Adv	Dulbecco’s Modified Eagle Medium Advanced
EFSA	European Food Safety Authority
EMA	European Medicines Agency
FBS	foetal bovine serum
FDA	Food and Drug Administration
FoxO3a	Forkhead box protein O3
HMGR	HMG-CoA reductase
HNF1α	Hepatocyte Nuclear Factor 1 Alpha
HPLC-MS/MS	High-Performance Liquid Chromatography coupled with Tandem Mass Spectrometry
HPLC-DAD	High-Performance Liquid Chromatography with Diode Array Detection
JAK2/STAT3	Janus Kinase 2/Signal Transducer and Activator of Transcription 3
LDL	low-density lipoprotein
LDLr	LDL receptor
MTT	3-(4,5-dimethylthiazol-2-yl)-2,5-diphenyltetrazolium bromide
NAFLD	non-alcoholic fatty liver disease
PCSK9	Proprotein Convertase Subtilisin/Kexin Type 9
PMSF	phenylmethanesulfonylfluoride
PVDF	polyvinylidene fluoride
ROS	radical oxygen species
RYRF	Fermented red rice
SAC	S-allyl-cysteine
SDS-PAGE	sodium dodecyl sulfate-polyacrylamide gel electrophoresis
SREBP-2	Sterol Regulatory Element-Binding Protein 2
TEER	trans-epithelial electrical resistance
TFC	Total flavonoid content
TPC	Total polyphenol content

## Appendix A

**Table A1:** LC–MS/MS analysis of the main bioactive compounds in the MIX prepared according to the selected experimental ratios of the four extracts following the dose–response screening step. Polyphenols are reported as cumulative contributions from all the respective source extracts.

Bioactive Compound	Content in MIX (%)	Analytical Method	Source Extract(s)
SAC	0.09 ± 0.03	LC–MS/MS	Black garlic
Polysaccharides	3.12 ± 0.16	LC–MS/MS	*Gastrodia elata*
Polyphenols	4.09 ± 0.20	LC–MS/MS	All extracts
Flavonoids	0.34 ± 0.02	LC–MS/MS	*Primula veris*
Tannins	0.93 ± 0.05	LC–MS/MS	AMLA

**Table A2:** Bliss independence analysis on HepG2 cells viability.

Treatment	Expected Data	Observed Data	Interaction
MIX	−48.45%	−71.98%	Synergistic

**Table A3:** Bliss independence analysis on HMGR levels reduction (ELISA kit).

Treatment	Expected Data	Observed Data	Interaction
MIX	−46.20%	−83.32%	Synergistic

**Table A4:** Bliss independence analysis on LDL content.

Treatment	Expected Data	Observed Data	Interaction
MIX	−16.42%	−57.55%	Synergistic

**Table A5:** Bliss independence analysis on bile production.

Treatment	Expected Data	Observed Data	Interaction
MIX	25.36%	60.65%	Synergistic

**Table A6:** Bliss independence analysis on PCSK9 expression reduction.

Treatment	Expected Data	Observed Data	Interaction
MIX	−30.24%	−65.31%	Synergistic

**Table A7:** Bliss independence analysis on SREBP-2 levels reduction (ELISA kit).

Treatment	Expected Data	Observed Data	Interaction
MIX	−34.47%	−63.77%	Synergistic

## Appendix B

**Table A8:** Effects of different treatments on HMGR levels under high-glucose conditions in vitro. Raw data (ng/mL) associated with the graph shown in Figure 4A. Values are expressed as mean ± SD of five independent experiments performed in triplicate. Percentage reduction indicates the decrease vs. Control High Glucose.

Samples	HMGR (ng/mL) Mean ± SD	Reduction vs. Control High Glucose (%)
Control High Glucose	34.76 ± 1.18	
RYRF	26.29 ± 2.31	24.37
AMLA	24.60 ± 1.28	29.23
Black Garlic	31.94 ± 1.12	8.11
*Gastrodia elata*	32.09 ± 1.33	7.68
Primula	30.70 ± 0.48	11.68
MIX	5.83 ± 0.52	83.32

**Table A9:** Effects of different treatments on total cholesterol levels in HepG2 cells under high glucose conditions in vitro. Raw data (µg/µL protein) is associated with the graph shown in Figure 4B. Values are expressed as mean ± SD of five independent experiments performed in triplicate. Percentage change indicates the difference vs. Control Normal Glucose.

Samples	Total Cholesterol (µg/µL) Mean ± SD	% Change vs. Control Normal Glucose
Control Normal Glucose	20.04 ± 0.79	
Control High Glucose	34.00 ± 0.92	70
RYRF	23.20 ± 0.90	16.01
AMLA	25.27 ± 0.52	26.35
Black Garlic	27.49 ± 0.55	37.45
*Gastrodia elata*	26.53 ± 0.72	32.67
Primula	26.81 ± 1.05	34.07
MIX	13.82 ± 1.12	−30.91

**Table A10:** Effects of different treatments on LDL uptake in HepG2 cells under high glucose conditions in vitro. Raw data (µg/µL protein) is associated with the graph shown in Figure 4C. Values are expressed as mean ± SD of five independent experiments performed in triplicate. Percentage change indicates the difference vs. Control High Glucose.

Samples	LDL Content (µg/µL Protein) Mean ± SD	% Change vs. Control High Glucose
Control High Glucose	0.53 ± 0.04	
RYRF	0.52 ± 0.05	−1.37
AMLA	0.45 ± 0.06	−14.35
Black Garlic	0.51 ± 0.03	−3.43
*Gastrodia elata*	0.50 ± 0.05	−5.50
Primula	0.51 ± 0.03	−4.50
MIX	0.23 ± 0.10	−57.55

**Table A11:** Effects of different treatments on total bile acid production in HepG2 cells under high glucose conditions in vitro. Raw data (µg/µL protein) associated with the graph shown in Figure 5A. Values are expressed as mean ± SD of five independent experiments performed in triplicate. Percentage change indicates the difference vs. Control High Glucose.

Samples	Bile Acid (µg/µL Protein) Mean ± SD	% Change vs. Control High Glucose
Control High Glucose	0.680 ± 0.05	
RYRF	0.714 ± 0.07	5
AMLA	0.780 ± 0.13	14.82
Black Garlic	0.698 ± 0.05	2.62
*Gastrodia elata*	0.712 ± 0.09	4.75
Primula	0.717 ± 0.06	5.49
MIX	1.092 ± 0.10	60.65

**Table A12:** Effects of different treatments on PCSK9 expression in HepG2 cells under high glucose conditions in vitro. Raw data (PCSK9/β-actin ratio) associated with the graph shown in Figure 6A. Values are expressed as mean ± SD of five independent experiments performed in triplicate. Percentage change indicates the difference vs. Control High Glucose.

Samples	PCSK9/β-Actin Ratio Mean ± SD	% Change vs. Control High Glucose
Control High Glucose	0.74 ± 0.05	
RYRF	0.85 ± 0.06	15
AMLA	0.64 ± 0.05	−13.23
Black Garlic	0.70 ± 0.03	−5.25
*Gastrodia elata*	0.72 ± 0.07	−3.50
Primula	0.699 ± 0.05	−5.50
MIX	0.26 ± 0.12	−65.31
